# Comparative Emergence of Maribavir and Ganciclovir Resistance in a Randomized Phase 3 Clinical Trial for Treatment of Cytomegalovirus Infection

**DOI:** 10.1093/infdis/jiae469

**Published:** 2024-09-20

**Authors:** Sunwen Chou, Drew J Winston, Robin K Avery, Catherine Cordonnier, Rafael F Duarte, Shariq Haider, Johan Maertens, Karl S Peggs, Carlos Solano, Jo-Anne H Young, Joan Gu, Ginger Pocock, Genovefa A Papanicolaou

**Affiliations:** Division of Infectious Diseases, Oregon Health and Science University, Portland, Oregon, USA; Research and Development Service, Veterans Affairs Portland Health Care System, Portland, Oregon, USA; Los Angeles Medical Center, University of California, Los Angeles, California, USA; Division of Infectious Diseases, Johns Hopkins University, Baltimore, Maryland, USA; Haematology Department, Henri Mondor Hôpital, Assistance Publique-Hopitaux de Paris, and Université Paris-Est-Créteil, Créteil, France; Department of Haematology, Hospital Universitario Puerta de Hierro Majadahonda, Madrid, Spain; Juravinski Hospital and Cancer Center, Hamilton Health Sciences, Hamilton, Ontario, Canada; Haematology Department, University Hospitals Leuven, Katholieke Universiteit Leuven, Leuven, Belgium; Department of Haematology, University College London Hospitals NHS Foundation Trust, London, United Kingdom; Hematology Department, Hospital Clínico Universitario, University of Valencia, Valencia, Spain; Division of Infectious Disease and International Medicine, University of Minnesota, Minneapolis, Minnesota, USA; Takeda Development Center Americas, Inc, Cambridge, Massachusetts, USA; Takeda Development Center Americas, Inc, Cambridge, Massachusetts, USA; Infectious Disease Service, Department of Medicine, Memorial Sloan Kettering Cancer Center, New York, New York, USA

**Keywords:** antiviral drug resistance, antiviral therapy, cytomegalovirus, maribavir, valganciclovir

## Abstract

**Background:**

Among 547 patients receiving maribavir or valganciclovir for first-episode cytomegalovirus infection after hematopoietic cell transplant, the treatment response rate was 69.6% and 77.4% respectively. Development of maribavir and ganciclovir resistance was compared after receiving either drug.

**Methods:**

Viral mutations conferring drug resistance were analyzed in plasma DNA extracts at baseline and posttreatment.

**Results:**

Prior antiviral drug exposure was limited, with only 2 instances of baseline drug resistance detected. An equal number (n = 241) received valganciclovir or maribavir for at least 21 days (median, 55–56 days). Among them, drug resistance mutations were detected in 24 (10%) maribavir recipients at 35–125 days (median, 56 days) after starting therapy, including in 12 of 14 who experienced a viral load rebound while on therapy. Ganciclovir resistance mutations developed in 6 (2.5%) valganciclovir recipients at 66–110 days (median, 90 days). One maribavir recipient developed a novel UL97 gene mutation (P-loop substitution G343A) that conferred strong maribavir and ganciclovir resistance in vitro. Viral clearance was confirmed in 17 (74%) of 23 patients with emergent maribavir resistance after retreatment with an alternative CMV antiviral drug.

**Conclusions:**

After 3–8 weeks of therapy, maribavir resistance emerged earlier and more frequently than ganciclovir resistance but was usually treatable using alternative therapy.

**Clinical Trials Registration**. NCT02927067 (AURORA).

## Teaser

In a randomized trial of maribavir or valganciclovir for treatment of first-episode cytomegalovirus infection after stem cell transplantation, maribavir resistance emerged earlier and more frequently than ganciclovir resistance but was usually treatable using an alternative therapy.

Effective management of opportunistic human cytomegalovirus (CMV) infection is essential for successful outcomes of hematopoietic cell transplantation (HCT). Strategies include antiviral prophylaxis or preemptive treatment of asymptomatic infection [1]. Because of adverse effects of traditional CMV DNA polymerase inhibitor antiviral drugs such as ganciclovir and foscarnet, including myelotoxicity and nephrotoxicity, there has been a welcome for the development of alternative options with different viral drug targets and avoidance of these toxicities. Letermovir, a CMV terminase inhibitor, has been approved for prophylaxis [2], and maribavir, a viral UL97 kinase inhibitor, has been approved for treatment of refractory CMV infection with or without drug resistance [3]. In a more recent phase 3 randomized trial of maribavir and valganciclovir for treatment of first-episode CMV infection after HCT, the treatment response rate, as defined by confirmed CMV viremia clearance at week 8, for maribavir was 69.6% versus valganciclovir at 77.4%, although fewer maribavir recipients developed neutropenia leading to treatment discontinuation [4].

A concern about the newer CMV antiviral drugs is their lower genetic barrier to the development of drug resistance, as observed in vitro [5]. The in vivo correlates remain incompletely defined. For example, emergent letermovir resistance in prophylaxis trials was rare [6]; however, case reports document the repeated detection of high-grade resistance mutations in off-label use of letermovir for the treatment of active CMV infection [7]. A phase 3 trial of maribavir for the treatment of refractory CMV infection (with or without drug resistance) revealed emergent maribavir resistance in 26% of treated patients [8], but a direct comparison of incidence with standard polymerase inhibitors was prevented by a history of extensive prior exposure to these drugs and differences in duration of drug exposure. The more recent randomized phase 3 trial of maribavir and valganciclovir as preemptive treatment after HCT [4] offered a better opportunity to evaluate any differences in emergent drug resistance after treatment with either drug, because prior antiviral drug exposure was limited and patients received comparable durations of study drug. A drug resistance analysis of this trial is reported here.

## METHODS

### Study Population, Treatment, and Primary End Point

As described in the primary study publication [4], the phase 3 trial (AURORA, NCT02927067) involved 547 HCT recipients with first-episode CMV infection (without end-organ disease), who received study drug after 1:1 randomization to maribavir at 400 mg twice daily or valganciclovir at 900 mg twice daily, for a planned duration of 8 weeks with 12 weeks of follow-up. As part of the phase 3 trial, all patients/legal guardians provided written informed consent for treatment and monitoring, and the trial was approved by the institutional review board of each participating institution.

Serial plasma CMV DNA viral quantitation was performed using the COBAS AmpliPrep/TaqMan assay at a central laboratory. Viral clearance was defined as a reading of <137 IU/mL (lower limit of quantitation) in consecutive postbaseline samples, separated by 5 days. The primary efficacy end point in the study was defined as clearance of plasma CMV DNA at the end of study week 8, with no alternative antiviral drug given up to that point. CMV therapy given prior to study entry was recorded.

### Resistance Analysis Protocol

Genotypic assessment of drug resistance was performed by fluorescent dideoxy (Sanger) sequencing of nested polymerase chain amplification products of plasma DNA extracts. The entire coding sequences of CMV genes UL27, UL54, and UL97 were targeted for sequencing, using overlapping bidirectional sequencing primers (Supplementary Table 1), with sequence readouts aligned to reference strain AD169 [9, 10]. Sequencing chromatograms were visually inspected for validation of automated readouts of novel or unusual amino acid substitutions in the vicinity of a known drug resistance mutation (DRM) or previous sequencing artifacts [8]. Amino acid substitutions were classified as known DRMs, polymorphisms previously identified in specimens from untreated patients, and unknown sequence variants. The genotyping schedule included a baseline pretreatment sample in all cases, and posttreatment samples where the plasma viral load was >455 IU/mL at weeks 4, 8, 16, and 20, with additional sampling at premature discontinuation of treatment, recurrence of viremia after prior clearance, or >1 log rise in viral load while on treatment.

### Recombinant Phenotyping

The effect of specific viral mutations on antiviral drug susceptibility was evaluated by transfer of the mutation to a baseline bacterial artificial chromosome clone BD1 of CMV strain AD169 containing a secreted alkaline phosphatase reporter gene for viral quantitation, followed by determination of the drug concentration required to reduce the growth reporter signal by 50% (EC_50_) in ARPEp cells [11]. Resistance is defined as a drug EC_50_ > 1.9-fold increase over the wild-type control. Relative fitness of viral mutants was compared using growth curves determined over the week following an equal inoculum [12].

## RESULTS

### Prior Antiviral Exposure and Baseline Virologic Findings

A history of prior administration of valganciclovir, ganciclovir, foscarnet, or cidofovir was recorded for 154 (28%) of 547 patients, of whom only 8 had a cumulative exposure of ≥21 days (up to 98 days). Of these 8 patients, none developed emergent drug resistance, 4 each were randomized to maribavir and valganciclovir, and 3 were responders to each drug, a similar proportion of responders as in the overall study population [4]. There was no recorded prior use of maribavir in any study patient.

A baseline plasma CMV DNA load was recorded by the central laboratory for 545 of 547 study patients. For 272 maribavir recipients, the median load was 2042 IU/mL and for 273 valganciclovir recipients, the median load was 2076 IU/mL. Considering only patients who received ≥21 days of study drug, 240 maribavir recipients had a median baseline load of 2135 IU/mL, with 42 having a load of ≥9100 IU/mL, and 240 valganciclovir recipients had a median baseline load of 2013 IU/mL, with 44 having a load of ≥9100 IU/mL.

Baseline viral genotyping for the full genes UL27, UL54, and UL97 was available for 88%, 78%, and 92%, respectively, of 273 maribavir recipients and 85%, 84%, and 90%, of 274 valganciclovir recipients. Among 33 patients with baseline samples yielding no data for any gene, 31 had a baseline viral load of <700 IU/mL and the 2 remaining had loads of 1581 and 2310 IU/mL, respectively. Only 2 patients (randomized to valganciclovir) had a baseline DRM detected (UL97 C592G and UL54 K513T), and they were taken off study drug before day 18. The remaining 502 baseline viral variants consisted of known polymorphisms (38%) and 311 variants of unknown phenotype (Supplementary Table 2), of which 81% were detected in only a single sample, 29% detected only as mixtures with wild-type sequence, and 32 (10%) detected in patients randomized to maribavir as well as those randomized to valganciclovir. No variants were at codons known to be associated with drug resistance.

### Emergent Drug Resistance Mutations

Among patients who received less than 21 days of study drug, 1 of 32 maribavir recipients and 2 of 33 valganciclovir recipients had a known DRM detected after treatment. These DRMs had no plausible relationship to the short period of study drug exposure. The maribavir recipient developed UL97 C592G at 103 days, after stopping maribavir at 14 days and receiving 2 months of subsequent valganciclovir therapy. A valganciclovir recipient had a day 8 specimen (viral load 1154 IU/mL) with detected DRMs of UL97 C480R and T409M, both as mixtures with wild-type sequence, together with multiple other mixed base changes in UL97 on visual review of primary sequencing data. These DRMs were not detected in a subsequent specimen from day 22 (viral load 12883 IU/mL). Neither DRM has been reported as selected after valganciclovir treatment. Therefore, the day 8 specimen was requested to be reextracted and resequenced, resulting in no finding of T409M or C480R, suggesting genotyping artifacts in the original readouts. A second valganciclovir recipient developed UL97 L595S at day 140 after discontinuing study medication at day 16 and extensive subsequent exposure to foscarnet, ganciclovir, and valganciclovir.

**Figure 1.**
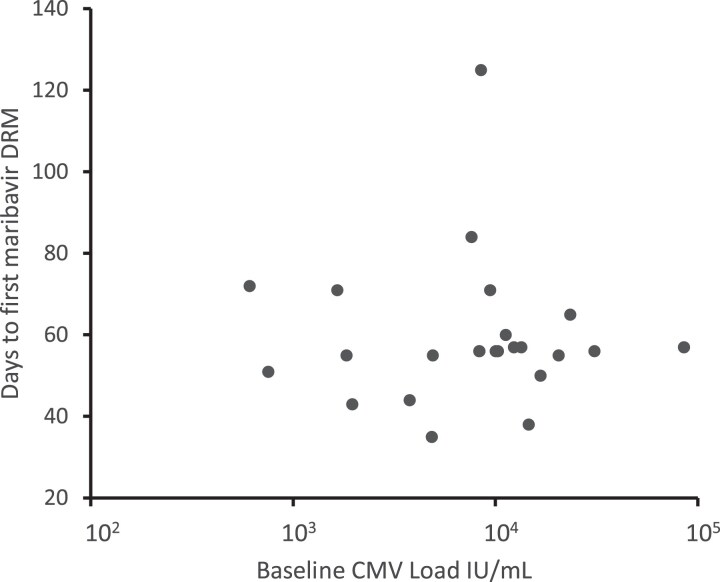
Baseline viral loads and interval to emergence of maribavir resistance. Each point represents the baseline CMV load and days to emergence of maribavir resistance for an individual patient. There is no correlation of the parameters (Pearson correlation coefficient −0.04). Abbreviations: CMV, cytomegalovirus; DRM, drug resistance mutation.

Table 1 and Table 2 show statistics on emerging drug resistance in those who received at least 21 days of study drug. Among 241 maribavir recipients, 24 (10%) developed a known UL97 maribavir DRM, detected at a median of 56 days after starting treatment. There was no correlation between the starting plasma CMV viral load and the interval to development of a maribavir DRM (Figure 1). However, the incidence of emergent maribavir resistance was higher (12 of 42, 29%) in those with a baseline plasma viral load of ≥9100 IU/mL than those with <9100 IU/mL (12 of 199, 6%) (Table 1). Available viral load data after 2 weeks of study drug (Table 2) indicate that those with a quantifiable load <1 log_10_ reduced from baseline (“refractory” as defined in another trial [3]) had a similarly higher incidence of emergent resistance, or higher with both risk factors present. Of 14 patients in the study who developed a rebound in viral load while on study drug after initial viral clearance, all were randomized to maribavir, and the 2 who did not have a DRM detected also did not have any posttreatment genotyping performed.

**Table 1.** Emergent Drug Resistance After ≥21 Days of Study Drug

Randomized Study Drug	Maribavir	Valganciclovir	*P* Value (Test)
Received ≥21 d, n	241	241	
Days of study drug treatment, median (range)	56 (21–62)	55 (21–63)	
Primary end point achieved, n (%)	187 (77.6)	210 (87.1)	.008^a^
Recurrence of CMV DNA while on therapy, n (%)	14 (5.8)	0	.0001^a^
Baseline plasma CMV DNA ≥9100 IU/mL, n	42	44	
Developed resistance mutation for study drug, n (%)	24 (10)	6 (2.5)	.001^a^
Days of study drug treatment, median (range)	56 (40–60)	55.5 (32–58)	
Days to detection of first DRM, median (range)	56 (35–125)	89.5 (66–110)	.007^b^
Recurrence of CMV DNA while on therapy, n (%)	12 (50)	0	
Baseline plasma CMV DNA ≥9100 IU/mL, n (%)	12 (50)	3 (50)	
Primary end point achieved, n (%)	4 (16.7)	4 (66.7)	.03^a^

Abbreviations: CMV, cytomegalovirus; d, days; DRM, drug resistance mutation.

^a^Fisher exact test (2-tailed).

^b^Student *t* test (2-tailed, unequal variances).

**Table 2.** Emergent Drug Resistance by Baseline Viral Load and Treatment Response at 2 Weeks

Study Drug and Subset	n	PrEndPt, n	PrEndPt, %	EDRM, n	EDRM, %
Maribavir	203	152	74.9	22	10.8
Baseline VL ≥9100 IU/mL	32	17	53.1	10	31.3
Refractory at 2 wk	67	33	49.3	20	29.9
Baseline VL ≥9100 IU/mL and refractory	19	6	31.6	9	47.4
Valganciclovir	194	167	86.1	5	2.6
Baseline VL ≥9100 IU/mL	33	23	69.7	2	6.1
Refractory at 2 wk	87	72	82.8	5	5.7
Baseline VL ≥9100 IU/mL and refractory	18	11	61.1	2	11.1

n is count of patients with treatment duration ≥21 days and available CMV load (VL) data at baseline and study day 14–18. Refractory is defined as CMV DNA quantifiable at <1 log reduction from baseline at 2-week visit (days 14–18).

Abbreviations: CMV, cytomegalovirus; EDRM, emergent drug resistance mutation for assigned drug detected posttreatment; PrEndPt, primary end point achieved; VL, viral load; wk, weeks.

Among 241 valganciclovir recipients, 6 (2.5%) developed a known DRM, a significantly lower percentage than maribavir recipients who developed a DRM (*P* = .001). Among those with a baseline viral load ≥9100 IU/mL, the incidence was 6.8% (3 of 44) (Table 1). A comparable incidence was observed with a refractory [3] virologic response at 2 weeks (Table 2). Other significant differences between valganciclovir and maribavir recipients who developed a DRM were the later timing of detection of ganciclovir DRM (in all cases after the end of scheduled study drug), and the proportion achieving the primary end point (4 of 24 for maribavir and 4 of 6 for valganciclovir). The median valganciclovir study drug duration was 55.5 days (range, 32–58 days); 4 of the 6 who developed a ganciclovir DRM had additional valganciclovir with or without ganciclovir exposure after discontinuation of study drug and before detection of the DRM.

Emergent DRMs are listed in Table 3, together with their maribavir and ganciclovir susceptibility phenotypes. Known DRMs detected after maribavir were mostly T409M alone (n = 11), H411Y alone (n = 5), or both (n = 7); 1 patient developed C480F together with T409M. DRMs detected after valganciclovir were among those typically associated with ganciclovir resistance [8, 11, 13]. Uncharacterized emergent variants (n = 45) in CMV genes UL27, UL54, and UL97 are listed in Supplementary Table 3. All but 1 were detected in a single patient. Based on emergence after at least 21 days of treatment, an associated viral load of >2000 IU/mL, failure to achieve primary end point, and proximity to known DRMs, UL97 substitution G343A was the only unknown variant with heightened suspicion of conferring drug resistance after exposure to the study drug. It was detected in 1 patient 54, 71, and 148 days after starting maribavir, in conjunction with UL97 H411Y on the first 2 occasions. As a novel UL97 P-loop mutation, G343A was selected for phenotypic evaluation of maribavir and ganciclovir resistance.

**Table 3.** Emergent Drug Resistance Mutations in UL97

Study Drug	Amino Acid Substitution	n^a^	Maribavir EC_50_ Ratio^b^	Ganciclovir EC_50_ Ratio^b^	Reference for EC_50_
Maribavir	G343A	1	**126**	**8.5**	Table 4
Maribavir	T409M	19	**80**	1.3	[11]
Maribavir	H411Y	12	**17**	0.7	[11]
Maribavir	C480F	1	**210**	**2.3**	[11]
Valganciclovir	M460I	1	*0.21*	**12**	[11]
Valganciclovir	M460V	1	*0.30*	**9.1**	[11]
Valganciclovir	H520Q	1	0.66	**9.7**	[11]
Valganciclovir	A594P	1	1.6	**7.9**	[11]
Valganciclovir	A594V	2	1.9	**6.9**	[11]
Valganciclovir	C603W	1	1.2	**5.9**	[11]

EC_50_ ratios ≥2.0 are shown in bold, ratios ≤0.5 are in italic.

Abbreviation: EC_50_, drug concentration that reduces viral growth by 50%.

^a^Number of patients developing indicated amino acid substitution.

^b^EC_50_ of mutant virus/EC_50_ of wild-type control strain.

Among the 24 patients who developed maribavir resistance, 23 received an alternative follow-up treatment and 17 (74%) of them achieved confirmed CMV DNA clearance within 6 weeks, 1 did not, and the remaining 5 did not have virologic data at 1 month to determine a treatment outcome. The alternative treatments included valganciclovir (n = 16), ganciclovir (n = 6), and foscarnet (n = 8). Some patients received more than 1 of these treatments.

### Recombinant Phenotyping

The newly observed P-loop UL97 substitution G343A was transferred into a cloned control CMV strain derived from laboratory strain AD169, alone and in combination with H411Y as observed in the study patient. The maribavir and ganciclovir susceptibilities of the recombinant viruses are shown in Table 4. G343A conferred a higher level of ganciclovir resistance (8.5-fold) and much greater maribavir resistance (126-fold) than the adjacent substitution F342Y (Table 3) [8]. These levels of resistance are at least as high as those conferred by the most common DRMs (A594V and T409M) for each drug (Table 3). The G343A-H411Y dual mutant showed high-grade maribavir resistance as expected. Comparative growth curves (Supplementary Figure) showed that the G343A mutant was more growth attenuated than the F342Y mutant and similar to the C480F mutant, while the G343A-H411Y double mutant was even more growth attenuated.

**Table 4.** Genotypes and Phenotypes of Recombinant Viruses

Strain^a^	Genotype^b^	Maribavir EC_50_, µM				Ganciclovir EC_50_, µM			
		Mean	SD	n	Ratio^c^	Mean	SD	n	Ratio^c^
Control strains									
4200	WT	0.12	0.03	17	…	1.3	0.17	35	…
4207	C592G	…	…	…	…	4.2	0.84	24	**3.2**
4353	H411Y	2.1	0.58	7	**17**	…	…	…	…
New recombinants									
4568	G343A	15	3.2	18	**126**	11	2.3	31	**8.5**
4570	G343A + H411Y	33	6.2	12	**272**	10	2.3	13	**7.9**

Abbreviations: CMV, cytomegalovirus; EC_50_, drug concentration that reduces viral growth by 50%; n, number of replicate assays; SD, standard deviation of the EC_50_ values; WT, wild type.

^a^Serial number of recombinant CMV strain.

^b^UL97 amino acid substitution shown.

^c^Ratio = EC_50_ of mutant virus/EC_50_ of WT control. Bold indicates EC_50_ > 1.9 × WT control.

## DISCUSSION

In this randomized treatment trial for first-episode asymptomatic CMV infection after HCT, emergent maribavir resistance was detected in 10% of those who received at least 3 weeks and a median of 8 weeks of maribavir therapy, significantly more often than the 2.5% who developed ganciclovir resistance after receiving a comparable duration of valganciclovir treatment. The overall incidence of maribavir resistance in this study is lower than the 26% reported in a previous phase 3 trial of treatment for refractory CMV infection with or without drug resistance in transplant recipients [8], where the median starting viral load was higher and the patient category was considered to be at higher risk of treatment failure because of host factors. As before, a viral load rebound while on treatment after an initial response (encountered in this study with maribavir but not valganciclovir) suggests emerging drug resistance.

Viral UL97 T409M and H411Y substitutions were the 2 most frequent genotypic markers of maribavir resistance in both studies, but C480F occurred much more often in the earlier salvage treatment trial (n = 21) [8] than in the current study (n = 1). The appearance of C480F after maribavir as salvage treatment but not as preemptive treatment was also observed in phase 2 trials [12]. Hypothetically, more extensive prior antiviral exposure may have selected for imperceptible subpopulations of C480F because it confers low-grade ganciclovir resistance at a fitness cost. Unlike in the other phase 3 trial [8], no cases of UL97 F342Y emerged. However, 1 patient revealed an unusual substitution at an adjacent codon of the kinase P-loop (G343A) that was found to confer a greater degree of maribavir and ganciclovir resistance than F342Y. Substitution G343A involves the third of 3 conserved glycine residues that define the kinase P-loop [14], with impact on biological UL97 kinase function and growth fitness, thus explaining why its substantial cross-resistance to both ganciclovir and maribavir would not be selected in preference to canonical mutations for each drug that do not impair growth to the same degree.

The more frequent and earlier detection of maribavir DRM is consistent with in vitro data suggesting that resistance to the newer CMV antiviral drugs letermovir and maribavir emerges after fewer cell culture passages than for ganciclovir and foscarnet [5]. However, published in vitro data do not directly compare the emergence of maribavir and valganciclovir resistance under the same viral culture conditions and therapeutic drug concentrations. Results of a randomized trial such as this one, with limited prior antiviral exposure, comparable starting viral loads, and treatment durations, are the best evidence for a clinically significant difference in the viral genetic barrier to drug resistance of newer antivirals in comparison with valganciclovir. The 2.5% incidence of resistance after valganciclovir treatment is compatible with previously published clinical trial statistics for prophylactic and therapeutic use (1.8% to 3.6% incidence) [15, 16], considering that 4 of the 6 cases had additional exposure to valganciclovir beyond the study protocol duration of 8 weeks.

The different incidences of emergent drug resistance can explain the difference in the proportion of maribavir and valganciclovir recipients (69.6% and 77.4%, respectively) who achieved the primary treatment response end point [4], because emergent resistance was associated with 20 cases of maribavir nonresponse and 2 cases of valganciclovir nonresponse (Table 1). Data in Table 2 illustrate the amplifying effects of higher starting viral loads and suboptimal virologic response at 2 weeks. Maribavir resistance arguably played a major role in treatment failure in 12 cases where a viral load rebound while on therapy coincided with the detection of a canonical maribavir resistance mutation.

Viral DNA clearance was observed in most cases of emergent maribavir resistance after alternative therapy, typically valganciclovir or ganciclovir, illustrating the absence of ganciclovir cross-resistance of the most common maribavir DRMs. This is similar to findings in the phase 3 salvage treatment trial [8], suggesting that maribavir-resistant CMV can be expected to respond to standard therapy after genotypic screening to exclude the uncommon cross-resistant UL97 mutations (now including G343A).

Limitations of this study primarily relate to the frequency of and technology for monitoring the evolution of resistance mutations. Most of the maribavir resistance was observed by traditional Sanger sequencing at the time of treatment discontinuation, although with more frequent sampling and deeper sequencing technology than used in this study, it is possible that mutant subpopulations could have been detected some days earlier [17]. A quality control concern is that variant subpopulations, especially when encountered in amplified DNA products from specimens with low viral loads (below 2000 IU/mL) can be poorly reproducible on reprocessing the same sample, whether by Sanger sequencing or next-generation sequencing [6, 17], as redemonstrated in 1 sample in this study. Thus, mixed unknown variant subpopulations encountered in single specimens of low copy number should be validated not only by reinspection of sequencing data, but also by independent testing of the same or a follow-up specimen from the same person. Such data were not available for many of the variants listed in the Supplementary Tables. There were not enough serial virologic and genotypic data for individual patients that would assess longer-term outcomes of salvage therapy and the persistence of maribavir resistance mutations after treatment is discontinued. No data were available from assays of therapeutic drug levels or immunologic parameters that could influence outcomes. Although this trial appears relatively unaffected by prior exposure to CMV antivirals, there was still some use of traditional DNA polymerase inhibitors that may not be completely recorded and may potentially affect the incidence of ganciclovir resistance.

In conclusion, this randomized trial confirmed clinically significant drug resistance to be more common with maribavir than valganciclovir after 3–8 weeks of treatment for asymptomatic CMV infection at similar starting viral loads, consistent with a hypothesis of a lower viral genetic barrier to resistance developed from in vitro observations. This difference may account for the lower observed primary end point response rate with maribavir. In circumstances where maribavir is chosen for its more favorable adverse effect profile, this study shows that emergent resistance can still be manageable by timely genotypic monitoring and switching to alternative therapy as necessary. Existing information indicates that maribavir and ganciclovir should not be used in combination [18]. Validation of UL97 G343A as another unusual P-loop mutation conferring substantial maribavir-ganciclovir cross-resistance illustrates the need for continued vigilance for novel DRMs.

## Supplementary Data

Supplementary materials are available at *The Journal of Infectious Diseases* online (http://jid.oxfordjournals.org/). Supplementary materials consist of data provided by the author that are published to benefit the reader. The posted materials are not copyedited. The contents of all supplementary data are the sole responsibility of the authors. Questions or messages regarding errors should be addressed to the author.

## Supplementary Material

jiae469_Supplementary_Data

## Notes

***Acknowledgments.*** Justin Watanabe provided technical assistance with recombinant phenotyping. We thank Dr Steven Kleiboeker (Eurofins Viracor Clinical Diagnostics) for providing technical details of genotyping assays. Under the direction of the authors, Sarah Morgan, PhD, employee of Excel Medical Affairs (Fairfield, CT), provided assistance in manuscript formatting, proofreading, and document assembly, but not in the composition of its content. Takeda Development Center Americas, Inc., Cambridge, MA provided funding to Excel Medical Affairs for this assistance.

***Author contributions.*** S. C. contributed study conception and design. S. C., J. G., and G. P. performed acquisition of data. All authors were involved in the analysis and interpretation of the data, drafting the article and/or revising it critically for important intellectual content, and approved the final version to be published.

***Data availability.*** The clinical trials datasets, including redacted study protocol, redacted statistical analysis plan, and individual participants’ data behind the results reported in this article, will be available within 3 months from initial request to researchers who provide a methodologically sound proposal. The data will be provided after its deidentification, in compliance with applicable privacy laws, data protection, and requirements for consent and anonymization.

***Financial support*.** This work was supported by Takeda Development Center Americas, Inc., Cambridge, MA; National Institutes of Health (grant number R01-AI116635 to S. C.); and use of US Department of Veterans Affairs facilities and resources. G. A. P. was partially supported by the National Institutes of Health/National Cancer Institute Cancer Center Support Grant (grant number P30 CA008748).

***Potential***  *c**onflicts of interest***. D. J. W. reports research grants from Ansun Biopharma, Cidara, Symbio, Takeda; and consulting fees from Shire, ViroPharma. R. K. A. reports study/grant support (paid to their institution) from AiCuris, Astellas, AstraZeneca, Chimerix, Merck, Oxford Immunotec, Qiagen, Regeneron, and Takeda. C. C. reports study/grant support (paid to their institution) from Merck Sharp & Dohme, Takeda; consulting fees from Takeda; and honoraria (personal) from Takeda, Merck Sharp & Dohme. R. F. D. reports consulting fees (advisor honoraria) from Cidara, Gilead Sciences, Jazz Pharmaceuticals, Merck Sharp & Dohme, Omeros Corporation, Sobi; honoraria (speaker's bureau) from Bristol Myers Squibb, Gilead Sciences, Jazz Pharmaceuticals, Merck Sharp & Dohme, Omeros Corporation, Takeda; support for attending meetings and/or travel from Bristol Myers Squibb, Gilead Sciences, Jazz Pharmaceuticals, Kite Pharma, Merck Sharp & Dohme, Omeros Corporation, Pfizer; and receipt of equipment and materials from Roche Diagnostics. S. H. reports honoraria (advisory board) from Takeda. J. M. reports honoraria (speaker) and advisory board participation from Takeda; consulting fees from F2G, Gilead Sciences, Mundipharma, Pfizer; honoraria from F2G, Gilead Sciences, Mundipharma, Pfizer; support for attending meetings and/or travel from F2G, Gilead Sciences, Mundipharma; and data safety monitoring board or advisory board for Cidara, F2G, Gilead Sciences, Mundipharma, Takeda. K. S. P. reports honoraria (speaker) from Takeda. C. S. reports honoraria (speaker) from GSK, Merck, Pfizer; and support for attending meetings and/or travel to American Society of Hematology Annual Meeting from Gilead. J.-A. H. Y. reports trial reimbursement (paid to their institution) from AlloVir, Ansun, Chimerix, Takeda; advisory board for AlloVir; Editor in Chief (completed 30 June 2022) of Clinical Microbiology Reviews; and Associate Editor (unpaid) of Transplantation and Cellular Therapy. J. G. is an employee of Takeda. G. P. was an employee of Takeda at the time of study. G. A. P. reports research funding (paid to their institution) from Merck Sharp & Dohme; consulting fees from Symbio, Takeda; honoraria from Merck Sharp & Dohme; data safety monitoring board for AlloVir, Armata, Merck Sharp & Dohme, Takeda, Trellis Bioscience; and end point adjudication committee for Octapharma. S. C. reports no potential conflicts.
