# Supplementary material for: Comparative Emergence of Maribavir and Ganciclovir Resistance in a Randomized Phase 3 Clinical Trial for Treatment of Cytomegalovirus Infection
Source: J Infect Dis. 2024 Sep 20;231(3):e470–7. doi: 10.1093/infdis/jiae469 (PMC11911792; doi:10.1093/infdis/jiae469)
Supplement: jiae469_Supplementary_Data [file jiae469_supplementary_data.zip › 240826_MBV302_V2_SupplmentaryData.pdf]

Supplementary Table 1. Primer sequences for CMV genotyping

Oligonucleotide	Oligonucleotide sequence 5'-3'
<u>UL97 Gene PCR</u>	
Bp CMV PCR UL97 #1 F	CGAGCTGAAGGACACGGTAGA
Bp CMV PCR UL97 #1 R	CTGAGGCTGTAATCGCACA
Bp CMV PCR UL97 #2 F	GGTGCTCGAAGAAAACGACG
Bp CMV PCR UL97 #2 R	AGACGCCCCACATGATGATG
<u>UL27 Gene PCR</u>	
Bp CMV PCR UL27 #1 F	GTCAGCGAGTACGTGTTTTCG
Bp CMV PCR UL27 #1 R	CTTCAGATCGTGGACGTGTTTG
Bp CMV PCR UL27 #2 F	AGTACTTGCCAGAGATCAGTCG
Bp CMV PCR UL27 #2 R	AGCTTCCAGTATTCTACGTCGC
<u>UL54 Gene PCR</u>	
Bp CMV PCR 54a 760 F	GCTGGTGCTCCGTGAATCG
Bp CMV PCR 54a 1434 R	AATATACACCTTGGTGGCGGC
Bp CMV PCR 54b 1162 F	CTGCTGCTGGGCTTTATGC
Bp CMV PCR 54b 2001 R	GCCGACGCTGCTACTACTGTTACT
Bp CMV PCR 54c (CLIN UL54b) 1902 F	GGCGGCTATGTTTCAGATGTC
Bp CMV PCR 54c (CLIN UL54b) 2601 R	CGTTCCCACTACATAGTCTTCCTGAT
Bp CMV PCR 54d 2544 F	GCGCGGTTCATCAAAGACA
Bp CMV PCR 54d 3407 R	TCGTCCGTGCCATCAATC
Bp CMV PCR 54e (-69) F	ATCTAGGTGCTGCATGTGTA
Bp CMV PCR 54e 829 R	CGACCTCGATATCACAAGTC
Bp CMV PCR 54f 3088 F	GCGGTTTCTGGCGTATCTT
Bp CMV PCR 54f 4029 R	CCAACGACGTGAGCGAGTC
<u>UL97 Gene Sequencing</u>	
Bp CMV SEQ UL97 #1-1 F	GACAGTGTCGGTGTGGTAGCT
Bp CMV SEQ UL97 #1-2 F	AGGGAAGACTGTCGCCACTA
Bp CMV SEQ UL97 #1-3 F	TGGATGCGCGAAGCTGC
Bp CMV SEQ UL97 #1-1 R	CGTCGTTTTCTTCGAGCACC
Bp CMV SEQ UL97 #1-2 R	GCCGTGGCGGATTCCTC
Bp CMV SEQ UL97 #1-3 R	GCTTCCACTGGTCGTGATGA
Bp CMV SEQ UL97 #2-1 F	AAAGTCAGGACAGCGCCGT
Bp CMV SEQ UL97 #2-2 F	GGACATGAGCGACGAGAGC
Bp CMV SEQ UL97 #2-3 F	GCTCATCGACGTGAACCCG
Bp CMV SEQ UL97 #2-4 F	GGCGTTATTGCATGTCGGAG
Bp CMV SEQ UL97 #2-1 R	GCGACACGAGGACATCTTGG
Bp CMV SEQ UL97 #2-2 R	AGACGCCCCACATGATGATG

Supplementary Table 1 (continued). Primer sequences for CMV genotyping

Oligonucleotide	Oligonucleotide sequence 5'-3'
UL27 Gene Sequencing	
Bp CMV SEQ UL27 #1-1 F	CGTCGTGAAGGAGGAGACTGTG
Bp CMV SEQ UL27 #1-2 F	ATGAACCCCGTGGATCAGC
Bp CMV SEQ UL27 #1-1 R	CGACTGATCTCTGGCAAGTACT
Bp CMV SEQ UL27 #1-2 R	AGCTCCTCGGTCATCGAGCATAG
Bp CMV SEQ UL27 #1-3 R	TGCGACCAGTCGGCGAA
Bp CMV SEQ UL27 #1-4 R	CTCGACGTTGCACGGGGTT
Bp CMV SEQ UL27 #2-1 F	GTGCACGTTGCTCGATTACAAG
Bp CMV SEQ UL27 #2-2 F	CTGTTTCTGGAGCCCGAGGA
Bp CMV SEQ UL27 #2-3 F	TCAGGCTGTTTAAAGGCGAGGC
Bp CMV SEQ UL27 #2-4 F	CAAACACGTCCACGATCTGAAG
Bp CMV SEQ UL27 #2-2 R	ATCGTCTAAATTCAAACCGCCG
UL54 Gene Sequencing	
Bp CMV SEQ 54a 779 F	TTACGACTGGCGGCAGC
Bp CMV SEQ 54a 1403 R	GGATTGTTGTGAGAAGCCGAGG
Bp CMV SEQ 54b 1381 F	CCTCGGCTTCTCACAACAATCC
Bp CMV SEQ 54b 1967 R	GGTGAAACGCCGTCCTGA
Bp CMV SEQ 54c (CLIN UL54b) 1948 F	CAGTCAGGACGGCGTTTCA
Bp CMV SEQ 54c (CLIN UL54b) 2567 R	GCAAAAAACACGGCTCTGAAA
Bp CMV SEQ 54d 2566 F	TTTCAGAGCCGTGTTTTTTGC
Bp CMV SEQ 54d 3227 R	TGCGGCAGGTTAGATTGACG
Bp CMV SEQ 54e (-61) F	GCTGCATGTGTATTTTCTTTGTGATT
Bp CMV SEQ 54e 795 R	CTGCCGCCAGTCGTAAC
Bp CMV SEQ 54f 3159 F	GAGGACCTGGTGCTTTCGT
Bp CMV SEQ 54f 3978 R	TGTGTCAGTCCAGCGGCA
Bp CMV SEQ 54e(2) 364 F	GCTATCGTCAGCATCTGGTG
Bp CMV SEQ 54a(2) 1180 R	GCATAAAGCCCAGCAGCAG

Supplementary Table 2. Baseline uncharacterized sequence variants

Study Med	Gene	Substitution	Count	Mix
MBV	UL27	P9L	1	
MBV	UL27	L11Q	2	
MBV	UL27	P16S	4	
MBV	UL27	H24R	1	Yes
MBV	UL27	R30H	1	
MBV	UL27	R46C	1	Yes
MBV	UL27	R64H	1	
MBV	UL27	A84T	4	
MBV	UL27	A85V	1	
MBV	UL27	G86S	1	
MBV	UL27	P98S	1	
MBV	UL27	H119R	1	Yes
MBV	UL27	P145Q	1	
MBV	UL27	R152C	1	
MBV	UL27	R178H	1	
MBV	UL27	R253Q	1	
MBV	UL27	A278S	1	Yes
MBV	UL27	D294S	1	Yes
MBV	UL27	N296del3	1	
MBV	UL27	N296G	1	
MBV	UL27	S303P	1	
MBV	UL27	S304P	1	
MBV	UL27	A309T	1	
MBV	UL27	A311G	2	
MBV	UL27	S315F	1	
MBV	UL27	A332T	1	Yes
MBV	UL27	A347V	1	
MBV	UL27	R348H	1	
MBV	UL27	D361N	1	Yes
MBV	UL27	A378V	1	
MBV	UL27	L436M	1	Yes
MBV	UL27	P514S	1	
MBV	UL27	R541Q	2	
MBV	UL27	A548V	1	
MBV	UL27	A572V	1	Yes
MBV	UL54	V11M	3	
MBV	UL54	K34R	1	
MBV	UL54	P36S	1	
MBV	UL54	M81I	1	
MBV	UL54	A139V	2	
MBV	UL54	R168H	1	
MBV	UL54	E169D	1	
MBV	UL54	P179L	1	Yes
MBV	UL54	P179S	1	
MBV	UL54	P206L	1	

Study Med	Gene	Substitution	Count	Mix
MBV	UL54	V245I	1	
MBV	UL54	D289N	1	Yes
MBV	UL54	R294C	1	Yes
MBV	UL54	P342L	1	
MBV	UL54	G344R	1	
MBV	UL54	D346N	1	
MBV	UL54	E353Q	1	
MBV	UL54	G354D	1	
MBV	UL54	Q397R	1	
MBV	UL54	V427L	1	
MBV	UL54	T437A	1	Yes
MBV	UL54	G451A	1	
MBV	UL54	Q455H	1	Yes
MBV	UL54	A459T	1	
MBV	UL54	S464C	1	
MBV	UL54	P468S	1	
MBV	UL54	T471M	1	
MBV	UL54	A529V	1	
MBV	UL54	A566G	1	Yes
MBV	UL54	R593H	1	
MBV	UL54	S602N	1	
MBV	UL54	T605M	1	
MBV	UL54	T610M	2	
MBV	UL54	V615A	1	
MBV	UL54	A636T	4	
MBV	UL54	P643S	2	
MBV	UL54	G659S	1	
MBV	UL54	S663G	1	
MBV	UL54	S664G	2	
MBV	UL54	S665del5	1	
MBV	UL54	F669V	1	
MBV	UL54	S670G	1	Yes
MBV	UL54	S682F	1	Yes
MBV	UL54	N685D	1	Yes
MBV	UL54	G687S	2	
MBV	UL54	A688S	1	
MBV	UL54	D711E	1	
MBV	UL54	P739S	1	Yes
MBV	UL54	T754A	1	
MBV	UL54	S782L	1	
MBV	UL54	R788C	1	
MBV	UL54	M791T	1	
MBV	UL54	E793D	1	
MBV	UL54	A849E	1	Yes
MBV	UL54	R850Q	5	Yes

Supplementary Table 2 (continued). Baseline uncharacterized sequence variants

Study Med	Gene	Substitution	Count	Mix
MBV	UL54	K853Q	1	Yes
MBV	UL54	N855K	2	
MBV	UL54	F866L	1	
MBV	UL54	D870E	1	Yes
MBV	UL54	D879N	1	
MBV	UL54	E882K	1	
MBV	UL54	S883I	2	
MBV	UL54	E888K	1	
MBV	UL54	G889R	1	Yes
MBV	UL54	S893L	1	
MBV	UL54	S894L	3	
MBV	UL54	G895E	1	
MBV	UL54	A928V	2	
MBV	UL54	R1006H	1	Yes
MBV	UL54	D1055E	1	
MBV	UL54	D1115N	1	Yes
MBV	UL54	D1118G	4	
MBV	UL54	S1119P	1	Yes
MBV	UL54	T1121I	1	
MBV	UL54	G1123D	2	
MBV	UL54	S1128L	1	
MBV	UL54	S1128P	1	
MBV	UL54	A1130T	1	Yes
MBV	UL54	A1130V	1	
MBV	UL54	D1132N	1	
MBV	UL54	D1132Y	1	
MBV	UL54	G1133D	1	
MBV	UL54	D1135G	1	
MBV	UL54	D1136E	1	
MBV	UL54	A1138V	1	
MBV	UL54	G1141S	1	
MBV	UL54	S1146G	5	
MBV	UL54	R1149I	1	
MBV	UL54	R1149K	1	
MBV	UL54	G1150E	1	
MBV	UL54	A1154T	1	
MBV	UL54	K1155del	2	
MBV	UL54	A1164T	1	
MBV	UL54	S1175N	1	
MBV	UL54	Y1176H	1	
MBV	UL54	V1197A	1	
MBV	UL54	V1200M	1	
MBV	UL54	E1209D	1	Yes
MBV	UL54	A1211T	1	
MBV	UL54	A1211V	1	

Study Med	Gene	Substitution	Count	Mix
MBV	UL54	H1218Y	1	
MBV	UL54	P1229Q	1	
MBV	UL97	A4T	1	
MBV	UL97	A12V	1	
MBV	UL97	T18I	1	
MBV	UL97	D72G	1	
MBV	UL97	D76G	1	
MBV	UL97	A78T	1	
MBV	UL97	T101A	1	
MBV	UL97	F102L	5	
MBV	UL97	V106L	1	Yes
MBV	UL97	G114R	1	
MBV	UL97	K116E	1	
MBV	UL97	D118G	1	
MBV	UL97	A119E	1	Yes
MBV	UL97	A119T	1	
MBV	UL97	A119V	4	
MBV	UL97	P129S	1	
MBV	UL97	G143S	1	
MBV	UL97	E161K	1	Yes
MBV	UL97	D167E	1	
MBV	UL97	D184N	1	Yes
MBV	UL97	S203P	1	Yes
MBV	UL97	R213C	1	
MBV	UL97	G216V	1	
MBV	UL97	R229H	2	
MBV	UL97	D234G	1	
MBV	UL97	G240D	1	
MBV	UL97	L248F	1	
MBV	UL97	S249N	1	
MBV	UL97	E254G	1	Yes
MBV	UL97	V260A	1	
MBV	UL97	T281I	1	
MBV	UL97	L387M	1	
MBV	UL97	V388M	1	
MBV	UL97	R394L	1	
MBV	UL97	V410I	1	Yes
MBV	UL97	V475M	1	Yes
MBV	UL97	A477V	1	Yes
MBV	UL97	A497V	1	
MBV	UL97	M526I	1	Yes
MBV	UL97	A648V	1	Yes
MBV	UL97	Y653C	1	
MBV	UL97	P706S	1	

Supplementary Table 2 (continued). Baseline uncharacterized sequence variants

Study Med	Gene	Substitution	Count	Mix
VGCV	UL27	P9S	1	
VGCV	UL27	L11Q	1	
VGCV	UL27	T13I	2	
VGCV	UL27	P16S	1	Yes
VGCV	UL27	D23E	1	
VGCV	UL27	G27S	1	
VGCV	UL27	E50K	1	Yes
VGCV	UL27	L82Q	1	Yes
VGCV	UL27	A84T	3	
VGCV	UL27	A85T	2	
VGCV	UL27	G86S	1	Yes
VGCV	UL27	G87D	1	Yes
VGCV	UL27	G87S	1	Yes
VGCV	UL27	A94T	1	
VGCV	UL27	P95S	2	
VGCV	UL27	H100Y	1	
VGCV	UL27	A114S	1	
VGCV	UL27	T128A	1	Yes
VGCV	UL27	H150Y	1	Yes
VGCV	UL27	G157D	1	Yes
VGCV	UL27	M212T	1	Yes
VGCV	UL27	H262N	1	
VGCV	UL27	T264M	2	
VGCV	UL27	N296D	2	
VGCV	UL27	N296insHDGN	1	
VGCV	UL27	G299D	1	
VGCV	UL27	S308P	1	Yes
VGCV	UL27	A311G	2	
VGCV	UL27	A311T	1	
VGCV	UL27	S312P	1	
VGCV	UL27	H329Y	1	
VGCV	UL27	A332T	1	Yes
VGCV	UL27	V353I	1	
VGCV	UL27	D369N	1	Yes
VGCV	UL27	A375V	1	Yes
VGCV	UL27	A442T	1	
VGCV	UL27	E468K	1	Yes
VGCV	UL27	D469N	1	Yes
VGCV	UL27	S490R	1	Yes
VGCV	UL27	P514L	1	Yes
VGCV	UL27	L549P	1	Yes
VGCV	UL54	T12I	1	
VGCV	UL54	G13C	1	
VGCV	UL54	G14S	1	

Study Med	Gene	Substitution	Count	Mix
VGCV	UL54	P26L	1	Yes
VGCV	UL54	P26S	2	
VGCV	UL54	H75Y	2	
VGCV	UL54	T90I	1	
VGCV	UL54	G93S	1	Yes
VGCV	UL54	Y156H	1	
VGCV	UL54	T193I	1	Yes
VGCV	UL54	T242S	1	Yes
VGCV	UL54	W263R	1	
VGCV	UL54	G267S	1	
VGCV	UL54	G307S	1	
VGCV	UL54	G347V	1	Yes
VGCV	UL54	R348Q	2	Yes
VGCV	UL54	G349S	1	
VGCV	UL54	C350Y	1	
VGCV	UL54	E353Q	1	Yes
VGCV	UL54	V355T	1	
VGCV	UL54	H359Q	1	
VGCV	UL54	V427M	1	
VGCV	UL54	A463T	1	Yes
VGCV	UL54	D594H	1	Yes
VGCV	UL54	G604S	3	
VGCV	UL54	T610M	1	
VGCV	UL54	A614T	1	
VGCV	UL54	V627M	1	Yes
VGCV	UL54	P628S	2	
VGCV	UL54	V634M	1	
VGCV	UL54	P642S	1	
VGCV	UL54	A647T	1	Yes
VGCV	UL54	A647V	1	Yes
VGCV	UL54	G659D	1	
VGCV	UL54	S673G	1	
VGCV	UL54	G687S	1	Yes
VGCV	UL54	A693V	1	Yes
VGCV	UL54	A766V	2	
VGCV	UL54	A786V	3	
VGCV	UL54	R850Q	1	Yes
VGCV	UL54	F851C	1	Yes
VGCV	UL54	I852V	1	
VGCV	UL54	N855K	1	
VGCV	UL54	D870E	1	Yes
VGCV	UL54	Y871S	1	
VGCV	UL54	G874K	1	
VGCV	UL54	T875M	1	

Supplementary Table 2 (continued). Baseline uncharacterized sequence variants

Study Med	Gene	Substitution	Count	Mix
VGCV	UL54	G878R	1	Yes
VGCV	UL54	D879N	2	
VGCV	UL54	S883I	2	
VGCV	UL54	S884I	1	
VGCV	UL54	G889R	1	Yes
VGCV	UL54	S894L	2	
VGCV	UL54	A928V	4	
VGCV	UL54	V1040L	1	
VGCV	UL54	S1059R	1	
VGCV	UL54	S1060R	1	
VGCV	UL54	A1084T	1	Yes
VGCV	UL54	G1105N	1	
VGCV	UL54	V1106insRTAPGV	1	
VGCV	UL54	T1108M	1	
VGCV	UL54	P1110S	2	
VGCV	UL54	N1116D	1	
VGCV	UL54	D1118G	1	Yes
VGCV	UL54	S1119P	2	
VGCV	UL54	T1121I	2	
VGCV	UL54	A1130V	1	Yes
VGCV	UL54	D1136N	2	Yes
VGCV	UL54	G1141S	1	
VGCV	UL54	S1146G	4	
VGCV	UL54	R1149K	1	
VGCV	UL54	P1153L	1	Yes
VGCV	UL54	A1154V	1	Yes
VGCV	UL54	K1156T	1	
VGCV	UL54	R1159Q	1	
VGCV	UL54	C1166R	1	Yes
VGCV	UL54	P1174S	1	Yes
VGCV	UL54	M1219L	1	Yes
VGCV	UL97	A4T	1	
VGCV	UL97	R6Q	1	
VGCV	UL97	A9V	1	
VGCV	UL97	T17M	2	
VGCV	UL97	Q51del3	1	
VGCV	UL97	V71L	1	Yes
VGCV	UL97	D72E	1	

Study Med	Gene	Substitution	Count	Mix
VGCV	UL97	L73del	1	
VGCV	UL97	G79S	1	
VGCV	UL97	T83A	2	
VGCV	UL97	T95A	1	
VGCV	UL97	L97F	1	
VGCV	UL97	H99Y	1	
VGCV	UL97	T101A	1	Yes
VGCV	UL97	F102L	4	
VGCV	UL97	E115K	1	Yes
VGCV	UL97	E117D	1	
VGCV	UL97	A119V	2	
VGCV	UL97	R127G	1	Yes
VGCV	UL97	P129S	2	
VGCV	UL97	V131del2	1	
VGCV	UL97	S133F	1	
VGCV	UL97	G138D	1	Yes
VGCV	UL97	A140V	1	Yes
VGCV	UL97	S187R	1	Yes
VGCV	UL97	R213C	1	Yes
VGCV	UL97	L221F	2	
VGCV	UL97	A238V	1	Yes
VGCV	UL97	G242V	1	
VGCV	UL97	Q246R	1	
VGCV	UL97	L248F	1	
VGCV	UL97	S251G	1	
VGCV	UL97	S251N	1	
VGCV	UL97	A257V	1	
VGCV	UL97	A262V	1	Yes
VGCV	UL97	S264F	1	Yes
VGCV	UL97	L295F	1	
VGCV	UL97	A327V	1	
VGCV	UL97	R374H	1	Yes
VGCV	UL97	P385S	1	Yes
VGCV	UL97	S386P	1	Yes
VGCV	UL97	D535N	1	
VGCV	UL97	T580A	1	Yes
VGCV	UL97	S642F	1	Yes
VGCV	UL97	A648V	1	Yes

Count = Number of patients with substitution detected. Mix = Detected as mix with wild type sequences

Supplementary Table 3. Uncharacterized Treatment Emergent Variants

Study Med	PrEndPt	Interval	Gene	Substitution
MBV	No	42	UL27	F55S
MBV	No	43	UL27	A85V
MBV	No	15	UL27	H112Y
MBV	No	29	UL27	L317V
MBV	No	21	UL54	T164S
MBV	Yes	124	UL54	S281F
MBV	Yes	95	UL54	G354S
MBV	No	43	UL54	T474I
MBV	No	56	UL54	R1037C
MBV	No	20	UL54	S1146G
MBV	No	13	UL54	G1150E
MBV	No	20	UL54	H1180Y
MBV	No	56	UL97	M111I
MBV	No	56	UL97	S232N
MBV	No	54	UL97	**G343A**
MBV	No	13	UL97	L372P
MBV	No	29	UL97	A543T
MBV	No	62	UL97	E575G
MBV	No	18	UL97	L608P
MBV	No	18	UL97	S642P
MBV	No	15	UL97	S657L
VGCV	Yes	112	UL27	P3L
VGCV	No	8	UL27	T92A
VGCV	No	54	UL27	Q149H
VGCV	Yes	139	UL54	T12A
VGCV	Yes	83	UL54	T12I
VGCV	Yes	7	UL54	D165G
VGCV	Yes	76	UL54	P293A
VGCV	Yes	24	UL54	G332E
VGCV	Yes	139	UL54	V337A
VGCV	Yes	112	UL54	Q397R
VGCV	No	54	UL54	A636T
VGCV	Yes	112	UL54	G687S
VGCV	Yes	78	UL54	A786V
VGCV	No	50	UL54	A786V
VGCV	Yes	139	UL54	S897F
VGCV	Yes	125	UL54	E949K
VGCV	Yes	7	UL54	A1109V
VGCV	Yes	112	UL54	A1138V
VGCV	No	6	UL54	R1149I
VGCV	Yes	112	UL54	R1149K
VGCV	No	7	UL54	G1150del
VGCV	No	54	UL97	G81del
VGCV	Yes	140	UL97	F102L
VGCV	Yes	78	UL97	D113N
VGCV	Yes	140	UL97	G143S

PrEndPt: Primary endpoint achieved
Interval: Days to emergence of mutation

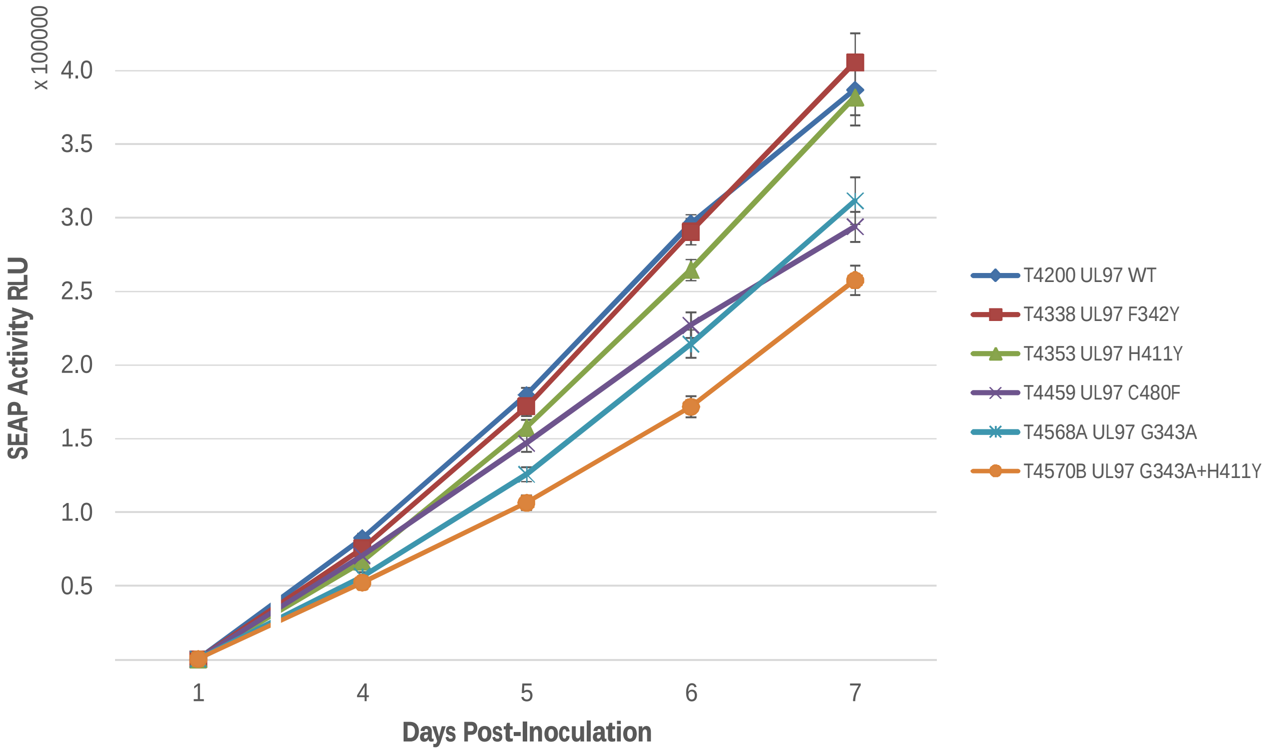

Supplementary Figure. Comparative growth curves of UL97 mutant strains

Viral stocks were inoculated into ARPEp cells at equivalent low multiplicity of infection as measured by culture supernatant SEAP value at 1 day. Serial samples of culture supernatants were collected at each of days 4 to 7 and assayed for SEAP activity as a measure of viral growth. Error bars denote standard error of the mean of 3 experiments set up in quadruplicate (total of 12 wells per strain).

SEAP = secreted alkaline phosphatase; RLU = relative light units; WT = wild type
